# Profile of patients in private home care who developed ventilator-associated pneumonia

**DOI:** 10.1590/0034-7167-2023-0146

**Published:** 2024-07-29

**Authors:** Fabiana Schimidt Cezar, Fabiana Camolesi Jacober, Heloísa Amaral Gaspar Gonçalves, Katia Vanessa Cantarini, Claudio Flauzino de Oliveira

**Affiliations:** I Home Doctor. São Paulo, São Paulo, Brazil

**Keywords:** Home Care Services, Pneumonia, Ventilator-Associated, Intensive Care Units, Health Systems Agencies, Respiration, Artificial, Atención Domiciliaria de Salud, Neumonía Asociada al Ventilador, Unidades de Cuidados Intensivos, Agencia Nacional de Vigilancia Sanitaria, Respiración Artificial

## Abstract

**Objectives:**

to analyze the profile and clinical outcomes of patients who developed Ventilator-Associated Pneumonia (VAP) in private home care and to compare the incidence with national data.

**Methods:**

this was a retrospective study with data collected from July 2021 to June 2022 from patient records at a private clinic. Patients using intermittent ventilation or without ventilatory support were excluded.

**Results:**

the utilization rate of mechanical ventilation was 15.9%. The incidence density of pneumonia in pediatrics was 2.2 cases per 1000 ventilation-days and in adults was 1.7 cases per 1000 ventilation-days, figures lower than those reported by the National Health Surveillance Agency. There were 101 episodes of pneumonia in 73 patients, predominantly male (65.8%), adults (53.4%), and those with neurological diseases (57.5%). The treatment regimen predominantly took place at home (80.2%), and there was one death.

**Conclusions:**

patients in home care showed a low incidence and mortality rate from ventilator-associated pneumonia.

## RESUMEN

**Objetivos:**

analizar el perfil y el desenlace clínico de pacientes que desarrollaron Neumonía Asociada a la Ventilación en Asistencia Domiciliaria privada y comparar la incidencia con datos nacionales.

**Métodos:**

estudio retrospectivo, con recolección de datos entre julio de 2021 y junio de 2022 en registros médicos de una clínica privada, excluyendo la ventilación intermitente y la ausencia de soporte ventilatorio.

**Resultados:**

la tasa de utilización de ventilación mecánica fue del 15,9%. La densidad de neumonía en pediatría fue de 2,2 casos por 1000 ventilaciones-día y en adultos de 1,7 casos por 1000 ventilaciones-día, datos inferiores a los reportados por la Agencia Nacional de Vigilancia Sanitaria. Se registraron 101 episodios de neumonía en 73 pacientes, en su mayoría varones (65,8%), adultos (53,4%) y con enfermedades neurológicas (57,5%). El régimen de tratamiento prevaleció en el domicilio (80,2%) y se registró un deceso.

**Conclusiones:**

los pacientes en Asistencia Domiciliaria presentaron baja incidencia y mortalidad por neumonía asociada a la ventilación mecánica.

## INTRODUCTION

Healthcare-Associated Infections (HAIs) are significant concerns within hospital settings, known to prolong hospital stays, worsen clinical outcomes, and increase care costs. Among HAIs, those related to devices such as mechanical ventilation, urinary catheters, and venous catheters receive particular scrutiny due to the severity and complexity of implementing effective prevention measures^(1-2)^.

Ventilator-Associated Pneumonia (VAP) is the leading cause of death from infections acquired within hospitals, exceeding complications associated with bloodstream infections, sepsis, and respiratory infections in non-ventilated patients^(3-5)^. The incidence density of VAP in North American hospitals varies from 0 to 4.4 cases per 1,000 ventilation-days^(6-7)^, while higher rates are observed in European centers. The EU-VAP/CAP study reported an incidence density of 18.3 cases per 1,000 ventilation-days^(8)^. Developing countries have also reported high rates, with 18.5 cases per 1,000 ventilation-days^(9)^. Brazilian data from 2020 show an incidence density of 10.6 cases per 1,000 ventilation-days in intensive care settings^(10)^. In the United States, each VAP case is estimated to result in an additional cost of $40,000 per hospitalization^(6,11)^.

The Home Care sector (HC) has experienced exponential growth over the past few decades in Brazil and globally, with an increasing number of patients and a rise in clinical complexity^(12-13)^. Home Care includes services for patients who require medication administration, enteral nutrition, wound care, rehabilitation therapies, oxygen therapy, and more complex therapies such as parenteral nutrition and Home Mechanical Ventilation (HMV)^(14)^. The increased clinical complexity of home-based patients is linked to the more frequent use of medical devices and ventilatory support, thus increasing the risk of VAP^(15)^.

Data on the incidence density of VAP in home settings are scarce but vital for monitoring service quality and assessing the effectiveness of preventive measures implemented. According to Silva et al.^(16)^, the literature indicates that pneumonia is the primary cause of hospital readmissions among pediatric patients.

HMV is increasingly prevalent, and just as in hospital settings, preventing VAP is crucial for maintaining safe care. The home environment is recognized for offering advantages over hospitals in terms of patient safety, reducing infection-related risks, and decreasing the incidence of VAP^(17)^.

## OBJECTIVES

To analyze the profile and clinical outcomes of patients who developed Ventilator-Associated Pneumonia (VAP) in private home care and compare the incidence with national data.

## METHODS

### Ethical Aspects

The study was conducted in accordance with national ethical guidelines, under the Certificate of Presentation for Ethical Appraisal (CAAE) and was approved by the Research Ethics Committee of the Padre Bento Hospital Complex in Guarulhos, São Paulo. Patient consent was waived as this observational study only utilized information from the Electronic Health Records (EHR), without the use of biological material and without any alteration to or influence on the routine treatment of the research participants, thereby posing no additional risk or harm to their well-being. All data were handled, analyzed, and presented anonymously for descriptive purposes, adhering to all confidentiality and privacy guidelines and norms.

### Design, Period, and Location of the Study

This was a retrospective epidemiological observational study guided by the Strengthening the Reporting of Observational Studies in Epidemiology (STROBE) tool. It was conducted through the analysis of secondary data obtained from the EHR from July 2021 to June 2022, across all units of Home Doctor. Home Doctor is a private home care company with 27 nationally distributed units, headquartered in São Paulo.

### Population, Inclusion, and Exclusion Criteria

All EHRs of patients who used continuous Home Mechanical Ventilation (HMV) were included. Excluded were those who did not receive home ventilatory support during the study period or who used ventilation intermittently.

### Study Protocol

Infection cases were analyzed by the Home Infection Control Service, through the completion of a specific form detailing signs and symptoms generated in the EHR with each new prescription. Through this analysis, infectious cases meeting epidemiological criteria for VAP were identified. For the diagnosis of VAP in patients on HMV, objective criteria previously defined by the institution were utilized, based on those proposed by the Association for Professionals in Infection Control and Epidemiology^(18)^, the Centers for Disease Control and Prevention^(19)^, and ANVISA^(10)^ (Chart 1). Patients diagnosed with COVID-19 were not included in the study.

**Chart 1:** Diagnostic Criteria for Ventilator-Associated Pneumonia

Criterion 1	A chest X-ray demonstrating pneumonia or the presence of a new pulmonary infiltrate, opacification, or cavitation AND at least one of the following respiratory changes: fever, new onset or worsening cough; increased or worsening chronic sputum; pleuritic chest pain; altered or recently worsened breath sounds (crackles, rhonchi, wheezes, or bronchophony); increased respiratory rate (≥ 25 per minute); O2 saturation < 94% in room air or a decrease in baseline O2 saturation of more than 3%.
Criterion 2	At least one of the following signs and symptoms must be present: fever with no other known cause; leukopenia or leukocytosis AND at least one of the following: emergence of purulent secretion or a change in the characteristics of the secretion, increased secretion, or increased need for aspiration; worsening gas exchange (deterioration of the PaO2/FiO2 ratio, increased oxygen needs, or increased ventilatory parameters).
Criterion 3	A patient with an underlying disease with two radiographs showing one of the following findings: new, progressive, and persistent infiltrate; opacification; cavitation.
Criterion 4	The patient must have at least two of the following signs and symptoms: fever; cough; appearance or increase in usual secretion; wheezing.

*O_2_ - Oxygen; PaO_2_ - Partial Pressure of Oxygen; FiO_2_ - Fraction of Inspired Oxygen.*

*Source: Home Doctor (2023).*

All patients on mechanical ventilation routinely receive a set of preventive measures adapted from those proposed by the Institute for Healthcare Improvement for hospitals^(20)^. These measures include hand hygiene, elevation of the head of the bed, and oral hygiene with 0.12% chlorhexidine.

The following data were collected from the Electronic Health Record (EHR) and exported to an Excel spreadsheet: utilization rate and duration of Home Mechanical Ventilation (HMV) until the development of Ventilator-Associated Pneumonia (VAP), VAP incidence density, gender, age group, age, diagnosis, treatment (location and antibiotic used), and outcome.

### Analysis of Results and Statistics

The utilization rate of continuous Home Mechanical Ventilation (HMV) was calculated by dividing the number of patients on HMV by the total number of patients treated during the period, then multiplying by 100.

The incidence density of Ventilator-Associated Pneumonia (VAP) was calculated using the following formula: the absolute number of VAP cases divided by the number of patient-days on HMV, multiplied by 1000. The incidence density of VAP in the study population was compared with the latest Brazilian intensive care data reported by ANVISA^(10)^ in 2020.

## RESULTS

During this period, Home Doctor treated 1,309 patients, of whom 1,050 were adults and 259 were pediatric. Of these, 208 (N: 102 adults and N: 106 pediatric) used Home Mechanical Ventilation (HMV). The overall utilization rate was 15.9%, with 9.7% among adult patients and 40.9% among pediatric patients. There were 101 episodes of Ventilator-Associated Pneumonia (VAP) between July 2021 and June 2022 in 73 patients, 14 of whom had repeated infectious episodes. The median duration of HMV use until the development of VAP was 245 days, with a standard deviation of 465 days.

**Figure 1:**
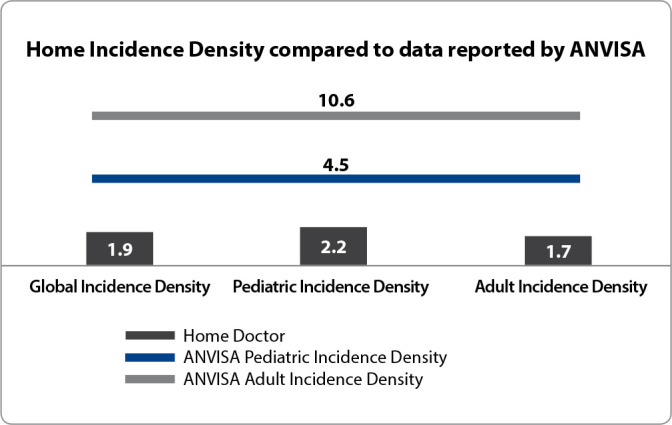
Comparison of the Incidence Density of Home Ventilator-Associated Pneumonia and the data reported by ANVISA in 2020 (N: 101)

*Source: ANVISA^(10)^, 2020.*

Figure 1 presents the comparison of the incidence density of VAP in home care with the data from Intensive Care Units (ICU) reported by ANVISA. The analysis reveals that the occurrence of VAP in ICUs is more than double in pediatrics and almost six times higher in adults when compared to the rates recorded at home.

This study found that among the 73 patients who developed one or more episodes of Ventilator-Associated Pneumonia (VAP), the majority occurred in male patients (65.8%) and adults (53.4%), with an average age of 57 years. The most prevalent diseases among patients with VAP who required continuous Home Mechanical Ventilation (HMV) were related to neurological pathologies (57.5%) (Table 1).

**Table 1:** Sociodemographic and clinical profile of patients with Ventilator-Associated Pneumonia on Home Mechanical Ventilation from July 2021 to June 2022. Home Doctor (N: 73)

Variables	n (%)	Mean (years)
Gender		
Female	25 (34.2)	
Male	48 (65.8)	
Age Group		
Adult	39 (53.4)	6.4±4
Pediatric	34 (46.6)	57±19
Diagnoses		
Central Nervous System Disorders	42 (57.5)	
Musculoskeletal System Disorders	8 (11.0)	
Circulatory System Disorders	6 (8.2)	
Infectious and Parasitic Diseases	5 (6.8)	
Respiratory System Disorders	4 (5.5)	
Other	8 (11.0)	

*N - Absolute number; Other - Congenital malformations, endocrine, nutritional and metabolic diseases, renal and gastrointestinal diseases.*

Of the 101 episodes of Ventilator-Associated Pneumonia (VAP), treatment predominantly occurred in the home setting; however, 20 patients (19.8%) were treated in a hospital environment, with an average hospital stay of 9.6 days. The most commonly used antibiotics for home treatment were Amoxicillin + Clavulanate (23.5%), Ceftriaxone (21.0%), and Meropenem (12.3%). There was one death (1%) in the home setting during the period analyzed (Table 2).

**Table 2:** Details of treatment and outcomes for patients with Ventilator-Associated Pneumonia. Home Doctor

Treatment	n (%)
Location	
Hospital	20 (19.8)
Home	81 (80.2)
Antibiotics (Home N: 81)	
Amoxicillin + Clavulanate	19 (23.5)
Ceftriaxone	17 (21.0)
Meropenem	10 (12.3)
Cefuroxime Axetil	7 (8.6)
Levofloxacin	6 (7.4)
Piperacillin + Tazobactam	5 (6.2)
Ertapenem	4 (5.0)
Other	13 (16.0)

*n - Absolute number; Other - Azithromycin, Ciprofloxacin, Gentamicin, Moxifloxacin, Amoxicillin, Amikacin, Cefepime, Clarithromycin, and Sulfamethoxazole + Trimethoprim.*

## DISCUSSION

Ventilator-Associated Pneumonia (VAP) in ICUs is a widely studied topic concerning the identification of risk factors, the definition and implementation of preventive measures, monitoring of incidence density, and the development of preventive and corrective actions, as well as the analysis of its financial and clinical impact. It is estimated that approximately 1 million patients per year are admitted to Home Care (HC) in Brazil, covering both public and private services^(21)^, with increasing use of Home Mechanical Ventilation (HMV), making VAP a concern in the home care setting.

Home Care is recognized for its psychosocial benefits compared to hospital care, allowing greater autonomy for the individual and the family. Moreover, it provides a unique care organization where the patient is naturally in physical isolation, without contact with other patients, and the health professional acts exclusively with one patient, which can contribute to the reduction of care-related risks and offers biological plausibility for the reduction of infection indicators compared to hospital settings^(22)^. The utilization rate of continuous HMV in adult patients found in this study was 9.7%, which is lower than the 41.5% rate reported by ANVISA^(10)^. In pediatrics, the utilization rate of HMV is similar to the rates in pediatric ICUs, with 40.9% and 39.1%, respectively.

The low incidences of VAP indicate safe and high-quality care. The reported density of VAP in Brazil in 2020 was 4.5 cases per 1,000 ventilation-days in pediatric ICUs and 10.6 cases per 1,000 ventilation-days in adult ICUs^(10)^. The current study showed 2.2 cases per 1,000 ventilation-days in pediatrics and 1.7 cases per 1,000 ventilation-days in adult patients, both of which are lower than the Brazilian epidemiological data from ICUs. This finding is corroborated by the study of Chenoweth et al.^(23)^, which analyzed a population of 57 patients receiving HMV and demonstrated a rate of 1.3 episodes of VAP per patient and a density of 1.5 cases per 1,000 ventilation-days. In contrast, Silva et al.^(16)^, analyzing 9 pediatric patients from January 2008 to June 2009, found 23 episodes of VAP and an average density of 6.8 cases per 1,000 ventilation-days, higher than hospital data, which may be attributed to their small sample size and the fragility of the implemented preventive measures.

The literature indicates a higher prevalence of hospital-acquired Ventilator-Associated Pneumonia (VAP) in adult male patients (71.4%), with an average age of 60.5 years^(24)^. In pediatrics, records show occurrences of VAP around the age of 4.5 years^(16)^. The demographic profile of VAP in this study aligns with that found in the literature, predominantly affecting male patients with an average age of 57 years and a prevalence of central nervous system pathologies (57.5%).

Regarding VAP treatment, the use of broad-spectrum antibiotic therapy is common in hospital settings^(11,25-26)^. In this study, the drugs used had a narrower spectrum, which suggests lower antimicrobial resistance in the home care environment. As for clinical outcomes, ICU data show that VAP developed in a hospital environment is severe, with mortality rates reaching up to 50% in various studies^(8,10,27-28)^. In contrast, this study reported a minimal home mortality rate from VAP.

### Study limitations

The results of this study should be interpreted considering its limitations. Although the data originate from a broad network of home care services, they may not accurately reflect patient conditions in other regions or different home care contexts. Comparing the small sample size of patients with Ventilator-Associated Pneumonia (VAP) to hospital literature might limit the statistical power of the comparisons and the ability to identify significant differences between groups. Additionally, the lack of similar studies hampers more effective comparisons. Future research should consider prospective and multicentric approaches to overcome these limitations and provide a more detailed understanding of the determinants of VAP in patients undergoing home mechanical ventilation.

### Contributions to the field

This work contributes by disseminating epidemiological data on patients in Home Mechanical Ventilation (HMV) who developed VAP at home, demonstrating the lesser complexity of this complication compared to hospital settings. Furthermore, it supports care and management teams for patients on mechanical ventilation in developing improvement plans aimed at reducing rates of home VAP as an indicator of good clinical practice.

## CONCLUSIONS

Home VAP has a lower incidence density than that reported by ANVISA for ICUs in 2020, demonstrating the capability of Home Care to treat patients with continuous HMV and low infectious complications. The home environment offers physical and social advantages that, in conjunction with preventive measures, are capable of reducing VAP incidence. The highest incidence of home VAP occurred in adult male patients diagnosed with neurological diseases. Patients with home VAP had the opportunity for treatment in their own environment, eliminating the need to travel to the hospital and the routine use of broad-spectrum antimicrobials, while also showing low mortality.
