# Supplementary material for: Profile of patients in private home care who developed ventilator-associated pneumonia
Source: Rev Bras Enferm. 2024 Jul 29;77(3):e20230146. doi: 10.1590/0034-7167-2023-0146 (PMC11290723; doi:10.1590/0034-7167-2023-0146)
Supplement: Translation

# Translation: Perfil de pacientes na assistência domiciliar privada que desenvolveram pneumonia associada à ventilação

**DOI:** 10.1590/0034-7167-2023-0146pt

**Authors:** Fabiana Schimidt Cezar, Fabiana Camolesi Jacober, Heloísa Amaral Gaspar Gonçalves, Katia Vanessa Cantarini, Claudio Flauzino de Oliveira

**Affiliations:** I Home Doctor. São Paulo, São Paulo, Brasil

**Keywords:** Assistência Domiciliar, Pneumonia Associada à Ventilação Mecânica, Unidade de Terapia Intensiva, Agência Nacional de Vigilância Sanitária, Ventilação Mecânica

## Abstract

**Objetivos:**

analisar o perfil e o desfecho clínico de pacientes que desenvolveram Pneumonia Associada à Ventilação em Assistência Domiciliar privada e comparar a incidência com dados nacionais.

**Métodos:**

estudo retrospectivo, com coleta entre julho de 2021 e junho de 2022 em prontuários de pacientes de uma instituição privada, sendo excluídos a ventilação intermitente e a ausência de suporte ventilatório.

**Resultados:**

a taxa de utilização de ventilação mecânica foi de 15,9%. A densidade de pneumonia na pediatria foi de 2,2 casos por 1000 ventilações-dia e em adultos de 1,7 casos por 1000 ventilações-dia, dados inferiores aos reportados pela Agência Nacional de Vigilância Sanitária. Ocorreram 101 episódios de pneumonia em 73 pacientes, em sua maioria do sexo masculino (65,8%), adultos (53,4%) e com doenças neurológicas (57,5%). O regime de tratamento prevaleceu no domicílio (80,2%) e ocorreu um óbito.

**Conclusões:**

os pacientes em Assistência Domiciliar apresentaram baixa incidência e mortalidade por pneumonia associada à ventilação mecânica.

## INTRODUÇÃO

As infecções relacionadas à assistência à saúde (IRAS) são assuntos relevantes no ambiente hospitalar, pois comprovadamente prolongam o tempo de internação, pioram o desfecho clínico e aumentam os custos da assistência. Dentre as IRAS, aquelas relacionadas a dispositivos (ventilação mecânica, cateter urinário e cateter venoso) são focos de atenção devido à gravidade e à complexidade da implementação de medidas efetivas de prevenção^(1-2)^.

A Pneumonia Associada à Ventilação (PAV) é a principal causa de óbito decorrente de infecções adquiridas no âmbito hospitalar, superando as complicações relacionadas a infecções de corrente sanguínea, sepse e infecções respiratórias em pacientes não ventilados^(3-5)^. As densidades de incidência de PAV em hospitais norte-americanos variam entre 0 a 4,4 casos/1.000 ventilações-dia^(6-7)^, enquanto que, nos centros europeus, há evidência de taxas superiores. O estudo EU-VAP/CAP relatou uma densidade de incidência de 18,3 casos/1.000 ventilações-dia^(8)^. Países em desenvolvimento também relatam taxas elevadas, 18,5 casos/1.000 ventilações-dia^(9)^. Dados brasileiros de 2020 reportam uma densidade de 10,6 casos/1.000 ventilações-dia em ambiente de terapia intensiva^(10)^. Nos Estados Unidos da América, estima-se um aumento de custo adicional de US$ 40.000 para cada internação que evoluiu com PAV^(6,11)^.

O setor de Assistência Domiciliar (AD) apresentou crescimento exponencial nas últimas décadas no Brasil e no mundo, com aumento progressivo do número e da complexidade clínica dos pacientes atendidos^(12-13)^. A AD inclui cuidados aos pacientes que necessitam de aplicação de medicamentos, nutrição enteral, realização de curativos, terapias de reabilitação, oxigenioterapia e terapias mais complexas como nutrição parenteral e Ventilação Mecânica Domiciliar (VMD)^(14)^. O aumento da complexidade clínica do paciente em domicílio está relacionado ao uso mais frequente de dispositivos médicos, bem como de suporte ventilatório, aumentando, desta forma, o risco de PAV^(15)^.

Os dados sobre a densidade de incidência domiciliar de PAV são escassos, porém cruciais para monitorar a qualidade do serviço prestado e definir a eficácia das medidas preventivas implementadas. Segundo Silva et al.^(16)^, a literatura reporta que a pneumonia é a principal causa de readmissões hospitalares em pacientes pediátricos.

A VMD vem se tornando cada vez mais prevalente e, assim como ocorre no ambiente hospitalar, a prevenção de PAV é um pilar para a manutenção de um atendimento seguro. Admite-se que o ambiente domiciliar apresente vantagens em relação ao hospital, no que tange à segurança do paciente, à redução dos riscos relacionados à infecção e à menor incidência de PAV^(17)^.

## OBJETIVOS

Analisar o perfil e o desfecho clínico de pacientes que desenvolveram Pneumonia Associada à Ventilação (PAV) em Assistência Domiciliar privada e comparar a incidência com dados nacionais.

## MÉTODOS

### Aspectos éticos

O estudo foi conduzido de acordo com as diretrizes de ética nacional, no âmbito do Certificado de Apresentação para Apreciação Ética (CAAE) e aprovado pelo Comitê de Ética em Pesquisa do Complexo Hospitalar Padre Bento de Guarulhos - CHPBG, no estado de São Paulo. O consentimento do paciente foi dispensado, por se tratar de um estudo observacional que empregou apenas informações do Prontuário Eletrônico do Paciente (PEP), sem utilização de material biológico e sem alterações ou influências na rotina/tratamento do participante da pesquisa, o que não adicionou riscos ou prejuízos ao bem-estar dos mesmos. Todos os dados foram manejados, analisados e apresentados anonimamente, para fins descritivos, cumprindo todas as diretrizes e normas de sigilo e confidencialidade.

### Desenho, período e local do estudo

Estudo observacional epidemiológico retrospectivo, norteado pela ferramenta *Strengthening the Reporting of Observational Studies in Epidemiology* (STROBE), realizado através da análise de dados secundários obtidos do PEP, entre julho de 2021 e junho de 2022, em todas as unidades pertencentes à *Home Doctor*. A *Home Doctor* é uma empresa privada de Assistência Domiciliar, composta por 27 unidades de abrangência nacional, com sede na cidade de São Paulo.

### População, critérios de inclusão e exclusão

Foram incluídos todos os PEPs referentes aos pacientes que utilizaram a Ventilação Mecânica Domiciliar (VMD) contínua, e excluídos aqueles que não receberam suporte ventilatório domiciliar durante o período estudado ou que utilizaram a ventilação de forma intermitente.

### Protocolo do estudo

Foram analisados os casos de infecção pelo Serviço de Controle de Infecção Domiciliar, mediante o preenchimento do formulário específico de sinais e sintomas gerados no PEP a cada nova prescrição. Por meio desta análise, foram identificados os casos infecciosos com critério epidemiológico de PAV. Para o diagnóstico de PAV em pacientes em VMD, foram utilizados critérios objetivos previamente definidos pela instituição, baseados nos propostos pela *Association for Professionals in Infection Control and Epidemiology* ^(18)^, *Centers for Disease Control and Prevention* ^(19)^ e ANVISA^(10)^ (Quadro 1). Não foram incluídos no estudo pacientes com diagnóstico de covid-19.

**Quadro 1:** Critérios diagnósticos de Pneumonia Associada à Ventilação Mecânica Domiciliar

Critério 1	Raio-X de tórax demonstrando pneumonia ou presença de novo infiltrado pulmonar, opacificação ou cavitação E pelo menos uma das seguintes alterações respiratórias: febre, tosse de início recente ou piora da tosse; expectoração ou piora de expectoração crônica; dor torácica tipo pleurítica; ausculta respiratória alterada ou com piora recente (estertores, roncos, sibilos ou broncofonia); aumento da frequência respiratória (≥ 25 por minutos); saturação de O_2_ < 94% em ar ambiente ou uma redução de saturação basal de O_2_> 3%.
Critério 2	Pelo menos um dos seguintes sinais e sintomas: febre sem outra causa conhecida; leucopenia ou leucocitose E pelo menos um dos seguintes: surgimento de secreção purulenta ou mudança das características da secreção ou aumento da secreção ou aumento da necessidade de aspiração; piora da troca gasosa (piora da relação PaO_2_ / FiO_2_ ou aumento da necessidade de oxigênio ou aumento dos parâmetros ventilatórios).
Critério 3	Paciente com doença de base com duas radiografias demonstrando um dos seguintes achados: infiltrado novo, progressivo e persistente; opacificação; cavitação.
Critério 4	Paciente tem pelo menos dois dos seguintes sinais e sintomas: febre; tosse; aparecimento ou aumento habitual da secreção; sibilos.

*O_2_ - Oxigênio; PaO_2_ - Pressão Parcial de Oxigênio; FiO_2_ - Fração Inspirada de Oxigênio.*

*Fonte: Home Doctor (2023).*

Todos os pacientes ventilados mecanicamente recebem rotineiramente um pacote de medidas preventivas adaptadas do proposto pelo *Institute for Healthcare Improvement* ^(20)^ para hospitais, que consistem em: higiene de mãos, elevação do decúbito e higiene oral com clorexidina 0,12%.

Foram coletados e exportados para planilha Excel os seguintes dados do PEP: taxa de utilização e tempo de VMD até o desenvolvimento de PAV, densidade de incidência de PAV, gênero, grupo etário, idade, diagnóstico, tratamento (local e antibiótico e desfecho).

### Análise dos resultados e estatística

A taxa de utilização de VMD contínua foi calculada através do número de pacientes em VMD pelo número de pacientes atendidos no período, multiplicado por 100.

Os dados de densidade de incidência de PAV foram calculados através da seguinte fórmula: número absoluto de PAV pelo número de pacientes em VMD-dia, multiplicado por 1000. A densidade de incidência de PAV da população do estudo foi comparada com últimos dados de terapia intensiva brasileira reportados pela ANVISA^(10)^ em 2020.

## RESULTADOS

A *Home Doctor* atendeu, neste período, 1309 pacientes, dos quais 1050 eram adultos e 259 pediátricos. Destes, 208 (N: 102 adultos e N: 106 pediátricos) utilizaram Ventilação Mecânica Domiciliar (VMD). A taxa de utilização global foi de 15,9%, sendo 9,7% entre os pacientes adultos e 40,9% entre os pediátricos. Ocorreram 101 episódios de Pneumonia Associada à Ventilação (PAV) entre julho de 2021 e junho de 2022 em 73 pacientes, dos quais 14 tiveram episódios infecciosos repetidos. A mediana de tempo de uso da VMD até o desenvolvimento de PAV foi de 245 dias, com desvio padrão de 465 dias.

A Figura 1 apresenta a comparação da densidade de incidência de PAV domiciliar com os dados de Unidade de Terapia Intensiva (UTI) reportados pela ANVISA. A análise revela que a ocorrência de PAV em UTIs é mais que o dobro em pediatria e quase seis vezes maior em adultos, quando comparada às taxas registradas no domicílio.

**Figura 1:** Comparação da Densidade de Incidência de Pneumonia Associada à Ventilação domiciliar e nos dados reportados pela ANVISA em 2020 (N: 101)

*DI - Densidade de Incidência; HD - Home Doctor.*

*Fonte: ANVISA^(10)^, 2020.*

Este estudo verificou que, dentre os 73 pacientes que desenvolveram um ou mais episódios de Pneumonia Associada à Ventilação (PAV), a maioria ocorreu em pacientes do sexo masculino (65,8%) e adultos (53,4%), com média de idade de 57 anos. As doenças mais prevalentes entre os pacientes com PAV que necessitaram de Ventilação Mecânica Domiciliar (VMD) contínua foram aquelas relacionadas a patologias neurológicas (57,5%) (Tabela 1).

**Tabela 1:** Perfil sociodemográfico e clínico dos pacientes com Pneumonia Associada à Ventilação em Ventilação Mecânica Domiciliar entre julho de 2021 e junho de 2022. *Home Doctor* (N: 73)

Variáveis	n (%)	Média (anos)
Gênero		
Feminino	25 (34,2)	
Masculino	48 (65,8)	
Grupo Etário		
Adulto	39 (53,4)	6,4±4
Pediatria	34 (46,6)	57±19
Diagnósticos		
Doenças Sistema Nervoso Central	42 (57,5)	
Doenças Sistema Osteomuscular	8 (11,0)	
Doenças Sistema Circulatório	6 (8,2)	
Doenças Infecciosas e Parasitárias	5 (6,8)	
Doenças Sistema Respiratório	4 (5,5)	
Outros	8 (11,0)	

*N - Número absoluto; Outros -* Malformações congênitas, doenças endócrinas, nutricionais e metabólicas, renais e gastrointestinais.

Dos 101 episódios de Pneumonia Associada à Ventilação (PAV), o tratamento ocorreu predominantemente no ambiente domiciliar; contudo, 20 pacientes (19,8%) foram tratados em ambiente hospitalar, com uma média de 9,6 dias de internação. Os antibióticos mais utilizados para o tratamento domiciliar foram Amoxicilina + Clavulanato (23,5%), Ceftriaxona (21,0%) e Meropenem (12,3%). Houve um óbito (1%) no período analisado em domicílio (Tabela 2).

**Tabela 2:** Detalhamento do tratamento e desfechos dos pacientes com Pneumonia Associada à Ventilação. *Home Doctor*

Tratamento	n (%)
Local	
Hospitalar	20 (19,8)
Domiciliar	81 (80,2)
Antibiótico (Domiciliar N: 81)	
Amoxacilina + Clavulanato	19 (23,5)
Ceftriaxona	17 (21,0)
Meropenem	10 (12,3)
Axetilcefuroxima	7 (8,6)
Levofloxacino	6 (7,4)
Piperacilina + Tazobactam	5 (6,2)
Ertpenem	4 (5,0)
Outros	13 (16,0)

*n - Número absoluto; Outros -* Azitromicina, Ciprofloxacino, Gentamicina, Moxifloxacino, Amoxicilina, Amicacina, Cefepima, Claritromicina e Sulfamatazol + trimetoprima.

## DISCUSSÃO

A Pneumonia Associada à Ventilação (PAV) em UTIs é um assunto amplamente estudado no que concerne à identificação de fatores de risco, à definição e implementação de medidas preventivas, ao acompanhamento da densidade de incidência e ao desdobramento de ações preventivas e corretivas, além da análise de seu impacto financeiro e clínico. Estima-se que aproximadamente 1 milhão de pacientes por ano sejam admitidos na Assistência Domiciliar (AD) no Brasil, abrangendo serviços públicos e privados^(21)^, com um crescente uso de Ventilação Mecânica Domiciliar (VMD), tornando a PAV uma preocupação no cenário domiciliar.

A AD é reconhecida por trazer benefícios nos aspectos psicossociais em relação ao hospital, permitindo maior autonomia do indivíduo e da família. Além disso, apresenta uma organização do cuidado diferenciada, onde o paciente está naturalmente em isolamento físico, sem contato com outros pacientes e o profissional da saúde atua exclusivamente em um paciente, o que pode contribuir para a redução dos riscos assistenciais e apresenta plausibilidade biológica no sentido de redução de indicadores de infecção, comparativamente ao hospital^(22)^. A taxa de utilização de VMD contínua em pacientes adultos encontrada neste estudo foi de 9,7%, inferior à taxa de 41,5% reportada pela ANVISA^(10)^. Em pediatria, a taxa de utilização de VMD assemelha-se às taxas das UTIs pediátricas, com 40,9% e 39,1% respectivamente.

As baixas incidências de PAV indicam um cuidado seguro e de qualidade. A densidade de PAV reportada no Brasil em 2020 foi de 4,5 casos/1000 ventilações-dia em UTI pediátrica e 10,6 casos/1000 ventilações-dia em UTI adulto^(10)^. O estudo atual demonstrou 2,2 casos/1000 ventilações-dia em pediatria e 1,7 casos/1000 ventilações-dia em pacientes adultos, ambas as densidades de incidência de PAV domiciliar são menores em relação aos dados epidemiológicos brasileiros de UTIs. Este achado é corroborado pelo estudo de Chenoweth et al.^(23)^, que analisou uma população de 57 pacientes recebendo VMD e demonstrou uma taxa de 1,3 episódios de PAV por paciente e densidade de 1,5 casos/1000 ventilações-dia. Em contraste, Silva et al.^(16)^, ao analisar 9 pacientes pediátricos no período de janeiro de 2008 a junho de 2009, encontraram 23 episódios de PAV e uma densidade média de 6,8 casos/1000 ventilações-dia, superior aos dados hospitalares, o que pode ser atribuído à sua pequena casuística e à fragilidade das medidas preventivas implementadas.

A literatura reporta maior prevalência de PAVs hospitalares em pacientes adultos do sexo masculino (71,4%), com uma média de idade de 60,5 anos^(24)^. Na pediatria, os registros apontam ocorrências de PAV por volta dos 4,5 anos^(16)^. O perfil demográfico de PAV neste estudo foi semelhante ao encontrado na literatura, predominando pacientes do sexo masculino e com média de idade de 57 anos, e uma prevalência de patologias do sistema nervoso central (57,5%).

Quanto ao tratamento de PAV, em ambiente hospitalar é comum o uso de antibioticoterapia de amplo espectro^(11,25-26)^. No presente estudo, as drogas utilizadas possuem menor espectro, o que reforça a menor resistência antimicrobiana no ambiente domiciliar. Em relação ao desfecho clínico, os dados de UTI demonstram que a PAV desenvolvida em ambiente hospitalar é grave, com mortalidade que pode atingir até 50% em diversas pesquisas^(8,10,27-28)^. Em contrapartida, neste estudo, a mortalidade domiciliar por PAV foi ínfima.

### Limitações do estudo

Os resultados deste estudo devem ser interpretados levando em conta as limitações. Embora os dados provenham de uma ampla rede de assistência domiciliar, eles podem não refletir precisamente as condições dos pacientes em outras regiões ou em diferentes contextos de cuidado domiciliar. Comparar o pequeno tamanho da amostra de pacientes com PAV à literatura hospitalar pode limitar o poder estatístico das comparações e a capacidade de identificar diferenças significativas entre os grupos. Por fim há ausência de estudos semelhantes para permitir melhores comparações. Pesquisas futuras devem considerar abordagens prospectivas e multicêntricas para superar essas limitações e proporcionar um entendimento mais detalhado dos determinantes de PAV em pacientes submetidos à ventilação mecânica domiciliar.

### Contribuições para a área

Este trabalho contribui divulgando dados epidemiológicos dos pacientes em VMD que evoluíram com PAV em domicílio, demonstrando a menor complexidade desta complicação quando comparada ao hospital. Além disso, apoia as equipes assistenciais e gerenciais dos pacientes em ventilação mecânica no que tange à elaboração de planos de melhoria visando a baixas taxas de PAV domiciliar como indicador de boas práticas assistenciais.

## CONCLUSÕES

A PAV domiciliar possui uma densidade de incidência menor do que a reportada pela ANVISA em relação às UTIs no ano de 2020, demonstrando a capacidade da Assistência Domiciliar em tratar pacientes com VMD contínua e com baixa complicação infecciosa. O ambiente domiciliar possui vantagens físicas e sociais que, em associação com medidas de prevenção, são capazes de promover a redução da PAV. A maior incidência de PAV domiciliar ocorreu em pacientes do sexo masculino, adultos e diagnosticados com doenças neurológicas. Os pacientes com PAV em domicílio tiveram a possibilidade de tratamento no próprio ambiente, sem a necessidade de deslocamento ao hospital e sem a utilização rotineira de antimicrobianos de amplo espectro, além de apresentarem baixa mortalidade.
